# *Clostridioides difficile para*-Cresol Production Is Induced by the Precursor *para*-Hydroxyphenylacetate

**DOI:** 10.1128/JB.00282-20

**Published:** 2020-08-25

**Authors:** Mark A. Harrison, Alexandra Faulds-Pain, Harparkash Kaur, Bruno Dupuy, Adriano O. Henriques, Isabelle Martin-Verstraete, Brendan W. Wren, Lisa F. Dawson

**Affiliations:** a Department of Infection Biology, London School of Hygiene and Tropical Medicine, London, United Kingdom; b Laboratoire Pathogenèses des Bactéries Anaérobies, Institut Pasteur, Université de Paris, Paris, France; c Instituto de Tecnologia Química e Biológica António Xavier, Universidade Nova de Lisboa, Oeiras, Portugal; d Université Paris Diderot, Sorbonne Paris Cité, Paris, France; University of Illinois at Chicago

**Keywords:** *Clostridium difficile*, *para*-cresol, tyrosine, *p*-HPA, reporter, SNAP tag, PhoZ, GusA, transcription, σ54, *Clostridioides difficile*, transcriptional regulation, transcriptional reporter

## Abstract

Clostridioides difficile is an etiological agent for antibiotic-associated diarrheal disease. C. difficile produces a phenolic compound, *para*-cresol, which selectively targets gammaproteobacteria in the gut, facilitating dysbiosis. C. difficile decarboxylates *para*-hydroxyphenylacetate (*p*-HPA) to produce *p*-cresol by the action of the HpdBCA decarboxylase encoded by the *hpdBCA* operon. Here, we investigate regulation of the *hpdBCA* operon and directly compare three independent reporter systems; SNAP-tag, glucuronidase *gusA*, and alkaline phosphatase *phoZ* reporters to detect basal and inducible expression. We show that expression of *hpdBCA* is upregulated in response to elevated *p*-HPA. *In silico* analysis identified three putative promoters upstream of *hpdBCA* operon—P_1_, P_2_, and Pσ^54^; only the P_1_ promoter was responsible for both basal and *p*-HPA-inducible expression of *hpdBCA*. We demonstrated that turnover of tyrosine, a precursor for *p*-HPA, is insufficient to induce expression of the *hpdBCA* operon above basal levels because it is inefficiently converted to *p*-HPA in minimal media. We show that induction of the *hpdBCA* operon in response to *p*-HPA occurs in a dose-dependent manner. We also identified an inverted palindromic repeat (AAAAAG-N_13_-CTTTTT) upstream of the *hpdBCA* start codon (ATG) that is essential for inducing transcription of the *hpdBCA* operon in response to *p*-HPA, which drives the production of *p*-cresol. This provides insights into the regulatory control of *p*-cresol production, which affords a competitive advantage for C. difficile over other intestinal bacteria, promoting dysbiosis.

**IMPORTANCE** Clostridioides difficile infection results from antibiotic-associated dysbiosis. *para*-Cresol, a phenolic compound produced by C. difficile, selectively targets gammaproteobacteria in the gut, facilitating dysbiosis. Here, we demonstrate that expression of the *hpdBCA* operon, encoding the HpdBCA decarboxylase which converts *p*-HPA to *p*-cresol, is upregulated in response to elevated exogenous *p*-HPA, with induction occurring between >0.1 and ≤0.25 mg/ml. We determined a single promoter and an inverted palindromic repeat responsible for basal and *p*-HPA-inducible *hpdBCA* expression. We identified turnover of tyrosine, a *p*-HPA precursor, does not induce *hpdBCA* expression above basal level, indicating that exogenous *p*-HPA was required for *p*-cresol production. Identifying regulatory controls of *p*-cresol production will provide novel therapeutic targets to prevent *p*-cresol production, reducing C. difficile’s competitive advantage.

## Abstract (precis)

Clostridioides difficile infection results from antibiotic-associated dysbiosis. *para*-Cresol, a phenolic compound produced by C. difficile, selectively targets gammaproteobacteria in the gut, facilitating dysbiosis. Here, we demonstrate that expression of the *hpdBCA* operon, encoding the HpdBCA decarboxylase which converts *p*-HPA to *p*-cresol, is upregulated in response to elevated exogenous *p*-HPA, with induction occurring between >0.1 and ≤0.25 mg/ml. We determined a single promoter and an inverted palindromic repeat responsible for basal and *p*-HPA-inducible *hpdBCA* expression. We identified turnover of tyrosine, a *p*-HPA precursor, does not induce *hpdBCA* expression above basal level, indicating that exogenous *p*-HPA was required for *p*-cresol production. Identifying regulatory controls of *p*-cresol production will provide novel therapeutic targets to prevent *p*-cresol production, reducing C. difficile’s competitive advantage.

## INTRODUCTION

Clostridioides difficile, previously classified as Clostridium difficile, is a major problem in health care systems and in the United States alone costs the economy up to $5.4 billion per year (1). C. difficile is a spore-forming bacillus, whose spores resist disinfectants, heat, and desiccation (2, 3), as well as surviving in hospitals and the environment providing a reservoir for infection. C. difficile spores are transmitted via the oral-fecal route (2); they germinate in the gut in response to bile acid germinants (4), when the natural protective gut microbiota has been disrupted with antibiotics (5). Infected patients are treated with either metronidazole, vancomycin, or fidaxomicin. However, relapse of C. difficile infection is a serious concern, with up to 20 to 30% of patients requiring multiple rounds of antibiotics and in extreme cases fecal microbial transplants to treat disease (6).

C. difficile produces two toxins, TcdA and TcdB, which damage the intestinal barrier via glycosyltransferase activities targeting Rho, Rac, and Cdc42 in the cytoskeleton (7, 8). However, other putative virulence factors have been identified (9). C. difficile is among four intestinal bacteria that produce *para*-cresol above 100 μM (10); *p*-cresol is an antimicrobial compound, which selectively targets Gram-negative bacteria in the host, reducing microbial diversity and increasing relapse in a murine model of infection (11). This selectively provides a competitive advantage for C. difficile over Gram-negative intestinal bacteria isolated from healthy human volunteers (11). Importantly, while 55 strains of intestinal bacteria from 152 species were shown to produce *p*-cresol, 51 of these strains produced between 10- and 1,000-fold lower levels than C. difficile (10, 12). The production levels of *p*-cresol unique to C. difficile combined with its microbiome altering effects make *p*-cresol production an important attribute for C. difficile during establishment of colonization and subsequent disease.

*p*-Cresol is produced from tyrosine fermentation with the intermediary formation of *p*-HPA, which is then decarboxylated by the HpdBCA decarboxylase to produce *p*-cresol (13). Disruption of any of the three genes of the *hpdBCA* operon encoding the decarboxylase renders C. difficile unable to produce *p*-cresol (14). Currently, very little is known regarding the genetic regulatory control of *p*-cresol production. Previously, we have shown that *p*-cresol production is inhibited in rich medium (brain heart infusion supplemented with yeast extract) and produced in less-rich medium (yeast peptone) (14). We can overcome the inhibition of *p*-cresol production in BHIS with the addition of the intermediate *p*-HPA (14), suggesting a novel regulatory mechanism of *p*-cresol formation.

There are a number of colorimetric and chemiluminescent reporters available for use in gene regulation studies (15). However, most transcriptional and translational reporters require the presence of oxygen to fully function and are therefore difficult to use in anaerobic bacteria (15, 16). Another complication is that C. difficile autofluoresces green, rendering the utilization of green fluorescent protein difficult (16). In recent years, a number of alternative reporters have been developed for use in C. difficile with various efficacies, including *phoZ* (15), SNAP and CLIP tags (17), and LOV domain (18), including iLOV and phiLOV (19), mCherryOpt (16), and FAST (20). SNAP-tag reporters, which are based on human *O*-6-methylguanine-DNA methyltransferase, have been successfully used in translational fusions to identify protein localization and for fluorescence microscopy (17, 21) but have received limited attention for quantification of promoter activity. Second, the glucuronidase reporter, *gusA*, from Escherichia coli has been used in a number of studies to assess expression of the C. difficile toxin genes; the promoter regions of *tcdA*, *tcdB*, and *tcdR* were fused to the E. coli *gusA* reporter gene and transferred into either Clostridium perfringens (22, 23) or E. coli (24) for analysis. More recently, the E. coli *gusA reporter* has been cloned with a constitutive (*cwp2*) or an inducible promoter (p*tet*) and assessed in C. difficile as a tool to compare the expression levels (25). Third, *phoZ*, an alkaline phosphatase from Enterococcus faecalis has been shown to be a cost-effective and easily quantifiable reporter for transcriptional studies in C. difficile (15). These three reporters represent the most promising reporters in the study of C. difficile and therefore warranted further investigation as well as direct comparison of their sensitivity, cost, and ease of method.

In this study, we investigate the regulation of expression of the *hpdBCA* operon encoding the HpdBCA decarboxylase, which converts *p*-HPA to *p*-cresol. Therein, we compare three different reporter systems (SNAP-tag, *phoZ*, and *gusA*) to determine their efficacy in the detection of basal and inducible expression. We detected low-level basal expression from the 402-bp region upstream of *hpdBCA* using all three reporters. Importantly, the *gusA* and *phoZ* reporters produced similar quantitative trends in expression from the *hpdBCA* promoter region, with a significant decrease in the induction ratio of the SNAP-tag in the presence of *p*-HPA, which was partially overcome by increasing the culture volume sampled. Second, we identify three putative promoters, P_1_, P_2_, and Pσ^54^, and show by site-directed mutagenesis that the P_1_ promoter alone is responsible for both basal and inducible expression of the reporter constructs. Furthermore, we identify that the turnover of tyrosine in minimal media to *p*-HPA is insufficient to significantly induce expression from the *hpdBCA* promoter region above basal level, suggesting that the conversion of tyrosine to *p*-HPA under these conditions may be inefficient. We found that operon expression was induced at a concentration of >0.1 to ≤0.25 mg/ml *p*-HPA. Finally, we have identified that *p*-HPA induced expression from the P_1_ promoter requires the presence of an inverted palindromic repeat (AAAAAG-N_13_-CTTTTT) located upstream of the P_1_ promoter.

## RESULTS

### Detecting expression from the *hpdBCA* promoter region using different transcriptional fusions.

**FIG 1:**
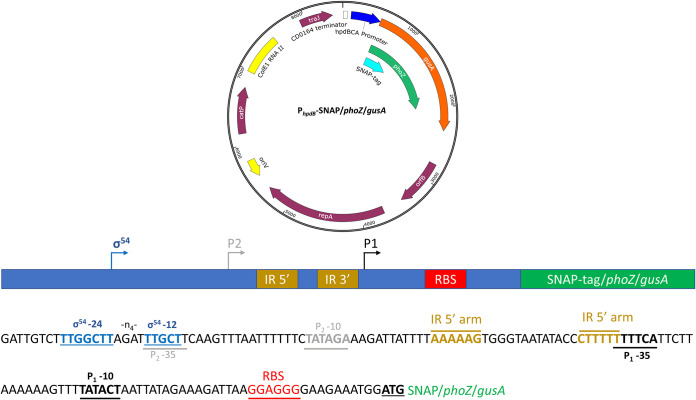
Overview of the SNAP, *phoZ*, and *gusA* reporter constructs. Each reporter was cloned into the C. difficile shuttle plasmid pMTL84151 and placed under transcriptional fusion with a 402-bp region (−399 to +3), including 399 bp directly upstream of *hpdBCA*, to the *hpdB* start codon (ATG).

Transcriptional control of the operon encoding the HpdBCA decarboxylase, which converts *p*-HPA to *p*-cresol, was assessed using three different reporter systems. A 402-bp DNA fragment corresponding to the promoter region located upstream of *hpdB* (−399 to +3) was fused, including the *hpdB* start codons (ATG) to the second codons of the SNAP-tag, *phoZ*, and *gusA* reporters, to produce in-frame reporter constructs (Fig. 1). Expression of the reporters was assessed in minimal medium (MM) in the presence or absence of *p*-HPA or tyrosine. We sought to detect and quantify basal and inducible expression from these reporters and to compare their efficiencies.

### SNAP-tag reporter fusion.

**FIG 2:**
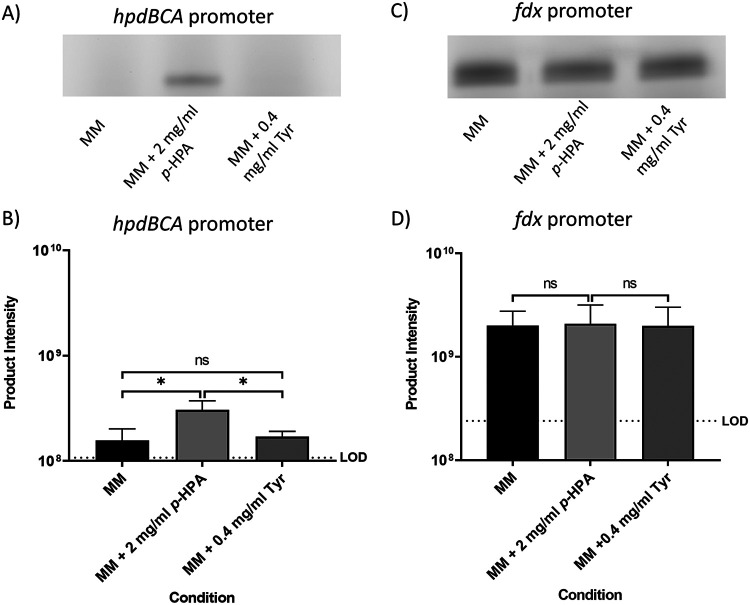
Visualization of the SNAP-tag reporter activity under the control of the *hpdBCA* promoter and a constitutive *fdx* promoter. Strains 630Δ*erm* P*_hpdB_*-SNAP (A and B) and 630Δ*erm* P*_fdx_*-SNAP (C and D) were grown for 4 h in MM, in MM plus 2 mg/ml *p*-HPA, or in MM plus 0.4 mg/ml tyrosine. Samples were run on an SDS-PAGE gel and processed with the fluorescent substrate TMR-Star prior to quantification of the SNAP-tag band using ImageQuant TL software. Three biological replicates were quantified using the following formula: product intensity = pixel volume/culture OD_590_. Dotted lines represent the limit of detection (LOD) calculated as the average of the results of the following formula for all samples: background pixel volume/culture OD_590_. Data represent means and standard errors. Statistical analysis was undertaken using linear regression to determine whether there is a significant effect of growth medium composition on the expression of the reporter construct in 630Δ*erm* P*_hpdB_*-SNAP (B) and 630Δ*erm* P*_fdx_*-SNAP (D). *, *P* < 0.05; ns, not significant. In panel D, no significant difference in expression was observed in MM alone compared to MM plus *p*-HPA and tyrosine.

A SNAP-tag reporter was fused to the *hpdBCA* promoter region (P*_hpdB_*-SNAP), as well as to a constitutive promoter *fdx* (derived from the ferrodoxin gene from Clostridium sporogenes NCIMB 10696) (P*_fdx_*-SNAP) in C. difficile (Fig. 2). The expression from these constructs was analyzed using SDS-PAGE as recommended by Cassona et al. (26) with additional gating of the fluorescent bands imaged by a Typhoon fluorescence imager for quantification. We assessed transcription in MM compared to MM with the addition of either 2 mg/ml *p*-HPA or 0.4 mg/ml tyrosine (Fig. 2). In the presence of *p*-HPA, transcription from the P*_hpdB_*-SNAP fusion was found to be significantly higher than that in MM (*P* = 0.036, coefficient of variance [COV] = 0.297) or MM plus 0.4 mg/ml tyrosine (*P* = 0.015, COV = 0.248) (Fig. 2A and B). The P*_fdx_*-SNAP control construct, with the SNAP-tag under the control of the constitutive *fdx* promoter, showed no significant difference in expression in minimal media compared to MM with the addition of *p*-HPA and tyrosine (Fig. 2C and D; see also File S1 in the supplemental material), therefore indicating that the effect of *p*-HPA on the induction of SNAP-tag production from the P*_hpdB_*-SNAP fusion was specific (Fig. 2).

### *phoZ* reporter fusion.

**FIG 3:**
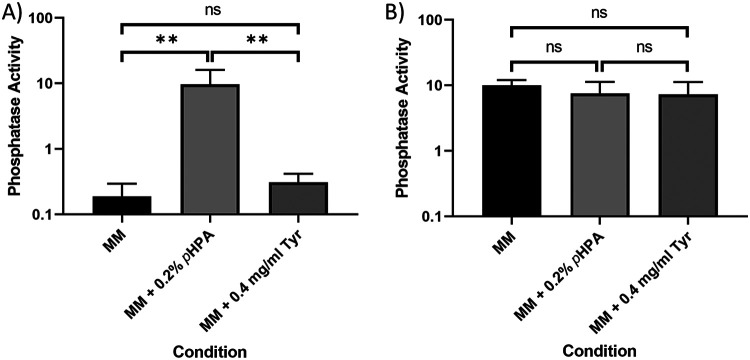
Visualization of the *phoZ* reporter activity under the control of the *hpdBCA* promoter and a constitutive *fdx* promoter. Strains 630Δ*erm* p*hpdBCA-phoZ* (A) and 630Δ*erm* P*_fdx_-phoZ* (B) were grown for 4 h in MM, in MM supplemented with 2 mg/ml *p*-HPA, or in MM supplemented with 0.4 mg/ml tyrosine. The expression of the *phoZ* reporters was quantified by using the following formula: [OD_420_ – (OD_550_ × 1.75)] × 1,000/*t* (min) × OD_590_ × volume of cells (ml). The data represent means and standard errors from three biological replicates. Statistical analyses were undertaken using linear regression to determine whether there is a significant effect on growth medium composition in 630Δ*erm* P*_hpdB_-phoZ* (A) and 630Δ*erm* P*_fdx_-phoZ* (B) strains (**, *P* < 0.01).

The promoter region of the *hpdBCA* operon was also fused to the chemiluminescent *phoZ* reporter gene (15). Using a P*_hpdB_-phoZ* fusion and a control P*_fdx_-phoZ* fusion (Fig. 3), we observed basal expression from the *hpdBCA* promoter when cells were grown in MM alone or in the presence of tyrosine, while the expression of the P*_hpdB_-phoZ* construct is induced in MM in the presence of *p*-HPA via detection of phosphatase activity (Fig. 3A). We noted that to optimize detection of promoter activity, the cultures harvested at 4 h required an overnight incubation with the *phoZ* substrate *p*-NPP to optimize detect basal level expression in MM and MM plus tyrosine. The presence of tyrosine gave a slight, but not significant increase in the expression of *phoZ* from the *hpdBCA* promoter region (Fig. 3A) (*P* = 0.207, COV = 0.237), while the addition of *p*-HPA significantly increased expression from the *hpdBCA* promoter region (Fig. 3A) (*P* < 0.01, COV = 1.69). We found no significant differences in expression of *phoZ* from the constitutive promoter (P*_fdx_*) under the different conditions tested (*p*-HPA comparison (*P* = 0.290, COV= −0.152) and tyrosine comparison (*P* = 0.330; COV = −0.187), suggesting that medium conditions had no effect on reporter detection and quantification using these methodologies (Fig. 3B) and that the effect of *p*-HPA was specific to the *hpdBCA* promoter.

### *gusA* reporter fusions.

**FIG 4:**
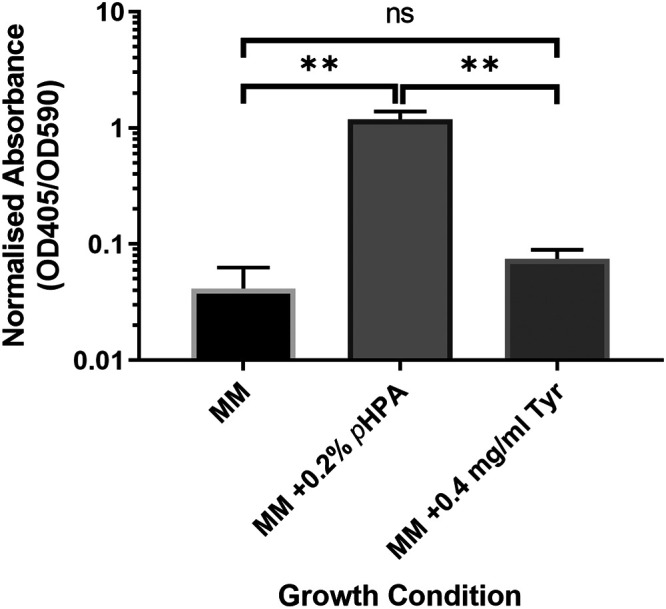
Visualization of the *gusA* reporter activity under the control of the *hpdBCA* promoter. 630Δ*erm* p*hpdBCA-gusA* was grown for 4 h in MM, in MM supplemented with 2 mg/ml *p*-HPA, or in MM supplemented with 0.4 mg/ml tyrosine. Expression was measured at OD_405_ and adjusted by growth using OD_590_. Data represent the means and standard errors from three biological replicates (**, *P* < 0.01). Statistical analysis was undertaken using linear regression to determine whether there is a significant effect on growth medium composition in the 630Δ*erm* P*_hpdB_-gusA*.

We also investigated whether *gusA*, an alternative chemiluminescent reporter to *phoZ*, could be used for such studies. Here, the same 402-bp upstream region of *hpdBCA* (−399 to +3) was cloned in a transcriptional fusion to *gusA*, including the *hpdB* start codon, to the second codon of *gusA* (P*_hpdB_-gusA*). We detected basal level expression from the P*_hpdB_-gusA* transcriptional fusion in MM, with a slight but not significant increase in the presence of tyrosine (Fig. 4) (*P* = 0.271, COV = 0.033). Once again, the addition of *p*-HPA resulted in a significant increase in expression from the *hpdBCA* promoter region (Fig. 4) (*P* < 0.01, COV = 1.143).

### Comparative quantification of fold change induction of the *hpdBCA* operon using different techniques.

When comparing reporter changes in the presence of *p*-HPA we see a (2.04 ± 0.61)-fold change when tested by the SNAP-tag (Fig. 2A), a (49.48 ± 8.71)-fold change by *phoZ* (Fig. 3A), and a (41.48 ± 23.35)-fold change by *gusA* (Fig. 4). Our analysis suggested that the SNAP-tag (Fig. 2) significantly underestimated the fold change compared to both the *phoZ* (*P* < 0.001, COV = −4.650) and *gusA* reporters (*P* = 0.002, COV = −4.234). Both *phoZ* and *gusA* reporters have the largest fold change of the three reporters in response to *p*-HPA, which may facilitate detection of small changes in expression.

### Induction of *hpdBCA* expression correlates with exogenous *p*-HPA.

**FIG 5:**
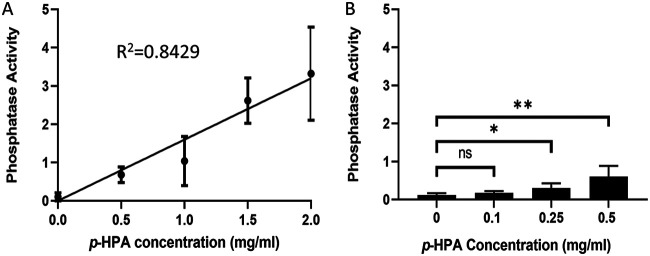
Use of the *phoZ* reporter to determine a concentration-dependent response to *p*-HPA for induction of the *hpdBCA* operon. (A) Expression from P*_hpdB_-phoZ* was monitored in MM with increasing concentrations of *p*-HPA (0.5, 1, 1.5, and 2 mg/ml). (B) Expression from P*_hpdB_-phoZ* was tested with *p*-HPA concentrations of 0.1, 0.25, and 0.5 mg/ml. The expression of the *phoZ* reporters was quantified by using the following formula: [OD_420_ – (OD_550_ × 1.75)] × 1,000/*t* (min) × OD_590_ × volume of cells (ml). Statistical analysis was performed. (A) Linear regression was used to determine any effect on phosphatase activity as *p*-HPA concentration increases (*R*^2^ = 0.8429, *P* < 0.001); (B) linear regression was used to determine at what *p*-HPA concentration there was a significant effect on expression compared to the absence of *p*-HPA (*, *P* < 0.05; **, *P* < 0.01).

Based on comparison of the fold change in response to *p*-HPA (Fig. 2A, 3A, and 4), we chose the P*_hpdB_-phoZ* fusion to examine the concentration-dependent induction of the *hpdBCA* promoter region in response to exogenous *p*-HPA. We showed a clear correlation between *p*-HPA concentration and *phoZ* expression (*R*^2^ = 0.8429, *P* < 0.001) (Fig. 5A). A significant induction of the P*_hpdB_-phoZ* fusion was detected in the presence of ≥0.25 mg/ml *p*-HPA (*P* = 0.025, COV = 0.406) (Fig. 5B).

### Inefficient turnover of tyrosine to *p*-HPA by *C. difficile*.

**FIG 6:**
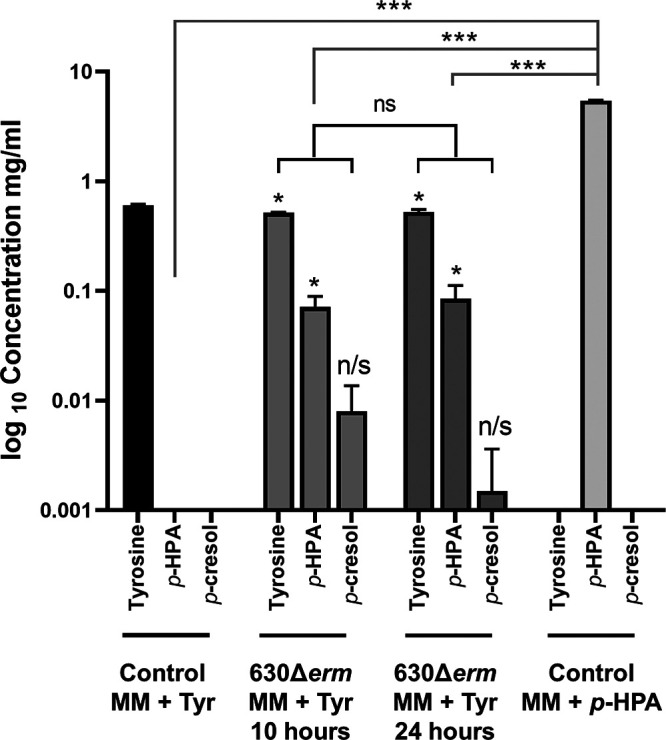
HPLC analysis to detect tyrosine, *p*-HPA, and *p*-cresol in spent culture media. The concentrations of tyrosine, *p*-HPA, and *p*-cresol in late-exponential-growth cultures of C. difficile strain 630Δ*erm* were determined by HPLC. Minimal medium was supplemented with 0.4 mg/ml tyrosine or 2 mg/ml *p*-HPA. Statistical analysis using linear regression was undertaken (i) to determine whether there was a significant increase in *p*-HPA and *p*-cresol production in media containing tyrosine (in black) and (ii) to determine whether there were any significant differences in the amount of *p*-HPA in the 2 mg/ml supplemented media compared to the amount of *p*-HPA produced in growth media containing tyrosine (in gray) (*, *P* < 0.05; ***, *P* < 0.001).

In MM supplemented with tyrosine, we used high-pressure liquid chromatography (HPLC) to determine the levels of tyrosine, *p*-HPA, and *p*-cresol after 10 h of growth (late exponential phase), in which we observed maximum C. difficile growth (measured as the optical density at 595 nm [OD_595_]), and 24 h of growth (stationary phase), at which we have previously observed maximum production of *p*-cresol (14). Here, we added the maximum soluble tyrosine concentration (0.4 mg/ml) to maximize the potential conversion of tyrosine to *p*-HPA. However, we saw no significant induction of expression of *hpdBCA* when tested by any of the reporters used in this study (Fig. 2 to 4). After 10 h, C. difficile utilized only 0.0895 mg/ml of the available tyrosine, leading to the production of 0.07 ± 0.02 mg/ml *p*-HPA (*P* = 0.031, COV = 0.0715), representing a turnover of 11.8% from tyrosine to *p*-HPA. This, in turn, led to a slight but not significant detection of *p*-cresol 0.008 ± 0.006 mg/ml (*P* = 0.106, COV = 0.008) (Fig. 6). No significant differences were observed in the concentrations of tyrosine, *p*-HPA and *p*-cresol between the 10- and 24-h time points (see File S1 in the supplemental material). Upon testing by the *phoZ* assay, no significant difference was detected in the expression of the P*_hpdB_-phoZ* fusion in the presence of 0.1 mg/ml *p*-HPA compared to no *p*-HPA; however, a significant increase in induction above baseline was detected at 0.25 mg/ml *p*-HPA (*P* = 0.025, COV = 0.406) (Fig. 5B). These results suggest that, under these conditions, tyrosine is very inefficiently used for the production of *p*-HPA and *p*-cresol. Theoretically, higher concentrations of tyrosine may induce more efficient turnover of tyrosine to *p*-HPA (by an as-yet-unidentified pathway) and therefore increase *p*-cresol production; however, tyrosine itself has no direct effect on the expression of the *hpdBCA* operon but only an indirect effect via the production of *p*-HPA.

### Regulation of *hpdBCA* expression.

**FIG 7:**
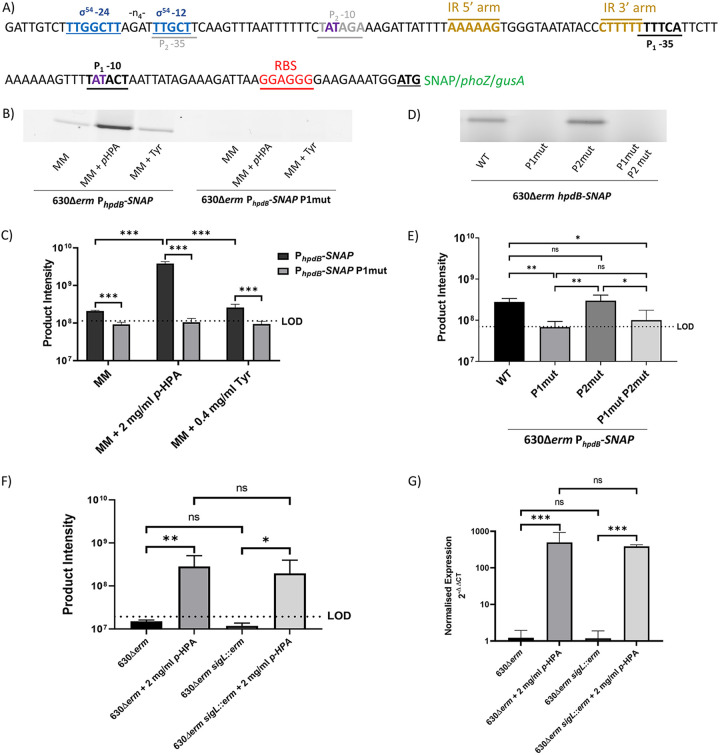
Mutation of the potential promoter regions in the *hpdBCA*-SNAP-tag reporter constructs. (A) Modifications by site-directed mutagenesis of the P_1_ and P_2_ promoter regions within the *hpdBCA*-SNAP reporter construct. The following putative promoter elements are marked on the diagram: putative ribosome binding site (red), putative P1 promoter (black), putative P2 promoter −10 region site (gray), putative SigL promoter (blue), putative inverted repeats (gold), and bases mutated by site-directed mutagenesis for functional analysis are indicated in purple. (B) SDS-PAGE gel image of the 630Δ*erm* P*_hpdB_*-SNAP alongside the mutated P_1_ promoter (630Δ*erm* P*_hpdB_*-SNAP P1mut). (C) Quantification of expression from 630Δ*erm* P*_hpdB_*-SNAP and 630Δ*erm* P*_hpdB_*-SNAP P1mut. (D) SDS-PAGE expression from the 630Δ*erm* p*hpdBCA*-SNAP compared to the mutated P_1_ promoter 630Δ*erm* P*_hpdB_*-SNAP P1mut, the mutated P_2_ promoter the 630Δ*erm* P*_hpdB_*-SNAP P2mut, and the mutation of both P_1_ and P_2_ promoters in MM plus 2 mg/ml *p*-HPA. (E) Quantification of expression from 630Δ*erm* p*hpdBCA*-SNAP compared to the mutated P_1_ promoter 630Δ*erm* P*_hpdB_*-SNAP P1mut, the mutated P_2_ promoter the 630Δ*erm* P*_hpdB_*-SNAP P2mut, and the mutation of both P_1_ and P_2_ promoters in MM plus 2 mg/ml *p*-HPA. (F) Strains 630Δ*erm* and 630Δ*erm sigL*::*erm* were transformed with P*_hpdB_*-SNAP construct. Expression of the SNAP-tag construct was assessed in BHISG medium compared to BHISG supplemented with 2 mg/ml *p*-HPA. Samples were run on an SDS-PAGE gel and processed with the fluorescent substrate TMR-Star prior to quantification of the SNAP-tag band using ImageQuant TL software. Three biological replicates were quantified using the following formula: product intensity = pixel volume/OD_590_. The dotted lines in panels C, E, and F the represent the limit of detection (LOD) calculated as the average of the following formula for all samples: background pixel volume/culture OD_590nm_. (G) qRT-PCR was used to assess the expression of *hpdC* in late exponential phase normalized to the 16S rRNA internal control (2^–ΔΔ^*^CT^*^)^). All data represent means and standard errors. Statistical analysis was undertaken using linear regression to determine (i) whether there is a significant effect of growth medium composition on expression from the P*_hpdB_*-SNAP in the 630Δ*erm* strain compared to the mutated P_1_ promoter P*_hpdB_*-SNAP P1mut, (ii) whether there is an effect on expression of P*_hpdB_*-SNAP in which the P_1_ and P_2_ −10 boxes were mutated (TAT>TGC bases) in the P_1_ and P_2_ −10 sites, (iii) whether there is a significant increase in expression of the SNAP construct in media supplemented with *p*-HPA in both 630Δ*erm* and 630Δ*erm sigL*::*erm* strains, and (iv) whether there are any differences in the expression of *hpdC* in the 630Δ*erm* strain compared to the 630Δ*erm sigL*::*erm* strain (*, *P* < 0.05; **, *P* < 0.01; ***, *P* < 0.001.

We identified three putative promoters upstream of the *hpdBCA* operon in the promoter region used to obtain transcriptional fusions. Two of them (P_1_ and P_2_) showed similarity to the consensus of the housekeeping sigma factor, SigA (TTGACA-N_17_-TATAAT) (27). The −10 box of the P_1_ promoter corresponds to the sequence TATACT, while the −35 box TTTTCA is located 16 nucleotides upstream of the −10 box (Fig. 7A). The P_2_ promoter contains −10 and −35 boxes TTGCTT-N_17_-TATAGA similar to the consensus of SigA-dependent promoters (Fig. 7A). A third putative promoter is present in the promoter region of the *hpdBCA* operon with a consensus with similarities to a σ^54^ (SigL) promoter site (TTGGCAT-N_5_-TTGCT) (28, 29). However, a reduced spacer between the −12 and −24 boxes (TTGGCTT-N_4_-TTGCT) (Fig. 7A) is present.

We first carried out site-directed mutagenesis to modify key bases TAT>TGC of the P_1_ promoter (Fig. 7A). Using a scaled-up SNAP-tag assay (see “Scaled-up SNAP-tag” below), we found improved performance since in the larger culture volume the fold change was found to be 18.48 ± 2.64 compared to the smaller culture’s fold change of 2.04 ± 0.61. We analyzed the effects of these mutations on basal and inducible expression of *hpdBCA* operon, in MM, MM with *p*-HPA, and MM with tyrosine (Fig. 7B and C). Mutation of the −10 box in the P_1_ promoter (TAT>TGC) abolished expression in MM (*P* < 0.001, COV = −0.357), MM supplemented with tyrosine (*P* < 0.001, COV = −0.440), and both basal and induced expression in the presence 2 mg/ml *p*-HPA (*P* < 0.001, COV = −1.574) (Fig. 7). In the presence of *p*-HPA, mutation of the P_2_ promoter (TAT>TGC) had no effect on expression from the *hpdBCA* promoter region (Fig. 7D and E) (*P* = 0.924, COV = −0.011), whereas the double mutation of both P_1_ and P_2_ led to no detectable expression (Fig. 7D). These results strongly suggest that P_1_ rather than P_2_ is functional.

To assess whether the putative SigL promoter had an effect on expression, we transferred the SNAP-tag reporter in a 630Δ*erm sigL*::*erm* background. After growth in BHIS (see Materials and Methods) supplemented with 100 mM glucose (BHISG), required by 630Δ*erm sigL*::*erm* for successful culture, in the presence or absence of *p*-HPA, we observed that *sigL* inactivation did not decrease expression of the SNAP reporter in the presence (*P* = 0.498, COV = −0.265) or absence (*P* = 0.071, COV = −0.107) of *p*-HPA (Fig. 7F). We confirmed by quantitative reverse transcription-PCR (qRT-PCR) that the expression of *hpdC* was unchanged in the *sigL*::*erm* mutant compared to 630Δ*erm* in BHISG broth (*P* = 0.943, COV = −0.121) and BHISG supplemented with *p*-HPA (*P* = 0.641, COV = 0.101) (Fig. 7G). These results confirm that the P_1_ promoter is solely responsible for expression and induction of *hpdBCA* operon under the conditions tested (Fig. 7). In addition, we found that by qRT-PCR fold change for 630Δ*erm* was 333.29 ± 169.26. In comparison to the qRT-PCR, all three of the reporters tested significantly underestimated fold change (see File S1 in the supplemental material). The relative fold change for the scaled-up SNAP-tag assay was 18-fold lower than the qRT-PCR, whereas the *phoZ* and *gusA* reporters were 6.8- and 8.2-fold lower, respectively, than the qRT-PCR.

### Response to *p*-HPA is dependent on the presence of an inverted repeat upstream of P_1_.

**FIG 8:**
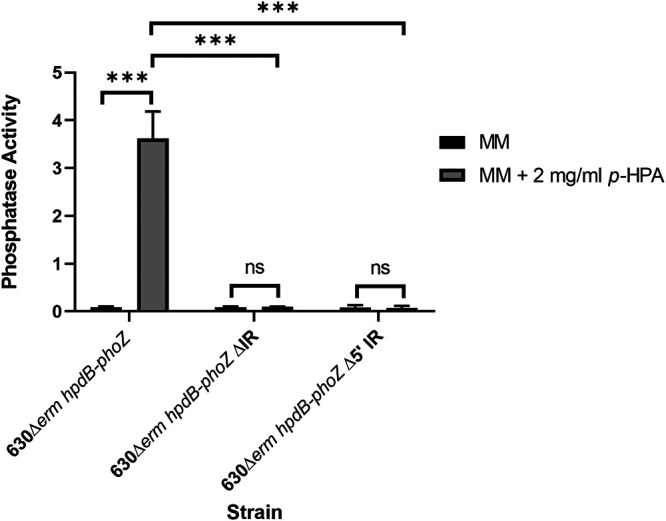
Mutation of the inverted repeat located within the *hpdBCA* upstream region leads to an inability to respond to *p*-HPA. Expression from the P*_hpdB_-phoZ* reporter was assessed in the presence or absence of an inverted repeat (AAAAAG-N_13_-CTTTTT) located 82 bp upstream of the *hpdB* start codon. Inverse PCR was used to delete the entire inverted repeat, including the spacer or the 5′ arm of the inverted repeat. Minimal medium was supplemented with 2 mg/ml *p*-HPA to determine whether any changes in expression were seen as a result of the mutation of the inverted repeat. ***, *P* < 0.001.

We sought to determine whether the presence of an inverted palindromic repeat (AAAAAG-N_13_-CTTTTT) overlapping the P_1_ −35 box was involved in induction of the P*_hpdB_-phoZ* fusion in response to *p*-HPA. Site-directed mutagenesis was used to remove either the 5′ arm of the inverted repeat or the entire inverted repeat from the P*_hpdB_-phoZ* fusion reporter. Removal of the 5′ arm (AAAAAG) or removal of the entire inverted repeat (AAAAAG-N_13_-CTTTTT) resulted in the abolition of inducible expression from the P*_hpdB_-phoZ* fusion in the presence of *p*-HPA (*P* < 0.001, COV = −1.015; and *P* = 0.003, COV = −1.303, respectively) (Fig. 8), without interfering with basal transcription, suggesting that this palindromic repeat is essential for responding to elevations in intrinsic or extrinsic *p*-HPA.

## DISCUSSION

Conditions that trigger *p*-cresol production by C. difficile have not been described to date. Here, we carried out a comparison of three different transcriptional reporters, as well as using them to ascertain the regulation of expression from the promoter region of the *hpdBCA* operon, which encodes the HpdBCA decarboxylase, responsible for the conversion of *p*-HPA to *p*-cresol. We demonstrate here that the *hpdBCA* operon has a single functional promoter (P_1_) that controls both basal expression and *p*-HPA induced expression. Moreover, we showed that an inverted repeat (AAAAAG-N_13_-CTTTTT), located 45 bp upstream of the P_1_ −10 site, seems to be essential for responding to the presence of *p*-HPA and inducing expression of the *hpdBCA* operon. Significant expression of *hpdBCA* above baseline is induced at a *p*-HPA concentration threshold in the range of >0.1 to ≤0.25 mg/ml.

Transcriptional regulation in bacteria is tightly regulated to facilitate temporal and spatial gene expression (30). There are limited direct comparisons between transcriptional reporters in anaerobic bacteria (31), particularly in C. difficile. We showed transcriptional activity and induction of the promoter region of *hpdBCA* using all three reporters (SNAP-tag, *phoZ*, and *gusA*). However, the methods of detection of the reporters vary, as do their sensitivities when using relatively small culture volumes. The SNAP-tag can be measured by spectrophotometry; however, to increase accuracy, we used SDS-PAGE gel separation and subsequent quantification. This mitigates the high-level background, visualized as a low-molecular-weight nonspecific fluorescent species (see Fig. S1 in the supplemental material). This method of quantification is more labor-intensive in comparison to the other two reporters. SNAP-tag assays are also amenable to being scaled up to facilitate detection of low-level transcripts. An increase in culture volume facilitated the detection of basal transcription from the *hpdBCA*-SNAP fusion and resulted in detection of induction ratio in comparable to the other reporters and qRT-PCR. One advantage of the SNAP-tag reporter is that multiple substrates can be used with a wide range of wavelengths and has been widely used in translational fusions to investigate protein localization via microscopy, including down to single cell detection (17), a feature that neither *gusA* nor *phoZ* reporters offer. The *gusA* and *phoZ* reporter assays are relatively simple, inexpensive, and easy to perform. In the present study, cost differences occur in processing of the harvested cells carrying the different reporters. This is largely due to the expense of the reporter substrates; per single assay, the cost was found to be approximately £10 for the SNAP-tag compared to under £0.20 for the *gusA* and *phoZ* reporters, respectively. Both the *phoZ* and the *gusA* reporters can be used in smaller culture volumes than the SNAP-tag and still detect basal expression. Each of the three reporters underestimated the fold change of induced expression compared to transcription detected by qRT-PCR, although both *phoZ* and *gusA* reporters outperformed the SNAP-tag. SNAP-tag performance was improved using a scaled-up method, which improved the sensitivity from 166-fold lower than the qRT-PCR to only 18-fold lower. The scaled-up SNAP-tag worked well for differentiating promoter deletions; however, this came at a cost of additional complexity and expense. A disadvantage of the *phoZ* reporter was that detection of basal expression required an overnight incubation, whereas both *gusA* and SNAP-tag reporters require a 30-min incubation in the presence of their substrates. Compared to the gold standard, qRT-PCR, we found that the *phoZ* reporter was the most efficient at detecting inducible expression while also being the most cost-effective system. It is possible in the future that new reporters will be more comparable to qRT-PCR in terms of detecting inducible expression.

Promoter consensus sequences have been identified for 9 of the 22 putative sigma factors in C. difficile, including SigA (27), SigH (27), SigL (σ^54^) (28), SigB (32), SigD (33), and SigK, SigE, SigG, and SigF (34). In the 399-bp region upstream of the *hpdB* start codon (ATG), we show that among the three potential promoters identified *in silico* using consensus previously identified, only P_1_ is functional and allows expression of the *hpdB* gene. The −10 box of P_1_ (TATACT) and −35 box of P_1_ (TTTTCA) have 83.3 and 66.7% homologies, respectively, to the consensus of σ^A^-dependent promoters. Mutation of the putative P_1_ −10 region (TAT>TCG) by site-directed mutagenesis abolishes expression, indicating that this promoter is crucial for *hpdBCA* expression, which is induced in the presence of *p*-HPA. Synthetic promoter biology has shown that the promoter (−10 and −35 sites), along with the spacer regions are essential for expression in clostridia (31). Expression of *hpdBCA* is regulated by an as-yet-unknown transcriptional regulator sensing *p*-HPA. In addition to identification of a single active promoter, we have shown that induction of this promoter by *p*-HPA is dependent on the presence of an inverted repeat located 82 bp upstream of the *hpdBCA* ATG directly upstream (AAAAAG-N_13_-CTTTTT). Indeed, removal of this inverted repeat abolished inducible expression from the promoter while retaining basal expression levels.

In agreement with previous findings by Selmer and Andrei (13), we show that tyrosine does not directly affect expression of the *hpdBCA* operon in our conditions. A limiting factor in this study is the solubility of tyrosine (0.4 mg/ml), which may be below a threshold needed to induce conversion of tyrosine to *p*-HPA, by an as-yet-unidentified pathway. However, under the conditions tested in this study, with the maximum soluble tyrosine concentration, the conversion of tyrosine to *p*-HPA is extremely low (0.07 mg/ml). Even if basal level expression from the *hpdBCA* operon was sufficient to detect low level turnover of tyrosine to *p*-HPA by HPLC, the concentration of *p*-HPA within the cell is likely insufficient to observe an induction. In contrast, expression of the *hpdBCA* operon can be induced as a direct response to exogenous *p*-HPA. In the presence of exogenous *p*-HPA, an increased turnover of *p*-HPA to *p*-cresol is observed. Interestingly, a recent study has identified a novel tyrosine transporter (35), which facilitates the uptake of tyrosine for utilization in the cell. However, although the tyrosine transporter mutants showed reduced *p*-cresol production, this transporter is not involved in the transport of *p*-HPA (35). This suggests the presence of a novel and currently unidentified *p*-HPA transporter.

We have shown previously that *p*-cresol inhibited growth of Gram-negative gut commensal bacteria at a concentration of 1 mg/ml (11). Here, we demonstrate turnover from tyrosine to *p*-HPA and *p*-cresol; however, in this system the turnover is inefficient and results in the production of 0.01 mg/ml *p*-cresol, which is unlikely to be physiologically relevant in terms of having a modulatory effect on the intestinal microbiome. However, we show that exogenous *p*-HPA can induce the *hpdBCA* operon promoting *p*-cresol production. Exogenous *p*-HPA is detected in fecal samples, which is suggestive of its presence within the human gut (36), albeit at unknown concentrations. A range of intestinal bacteria (11) and human cells (36) produce *p*-HPA, potentially providing C. difficile with exogenous *p*-HPA, which then induces *p*-cresol production at concentrations necessary to adversely affect the diversity of the microbiome. The gene expression profile of C. difficile greatly varies depending on the environment, with genes involved in metabolism particularly affected by environmental changes in the gut (reviewed by Theriot and Young [37]); therefore, it is reasonable to hypothesize that C. difficile is able to respond efficiently to the availability of exogenous *p*-HPA.

Our results demonstrate new insights into the regulation of *p*-cresol production in C. difficile, highlighting that the HpdBCA decarboxylase is produced from a single promoter P_1_, driving both basal and inducible expression of the *hpdBCA* operon. We have shown that *p*-HPA triggers expression of the *hpdBCA* operon in a concentration-dependent manner via an inverted repeat sequence directly upstream of the P_1_ promoter to enhance expression of the *hpdBCA* operon and upregulate *p*-cresol production. This strongly suggests that the *hpdBCA* operon is controlled by a positive uncharacterized regulator likely sensing *p*-HPA. A better understanding of the pathways leading to *p*-cresol production may help us develop strategies to inhibit *p*-cresol production and therefore reduce its deleterious effects on the microbiome.

## MATERIALS AND METHODS

### Bacterial strains and growth conditions.

C. difficile strains used in this study are listed in Table 1. C. difficile strains were routinely grown on brain heart infusion (Oxoid) supplemented with 5 g liter^−1^ yeast extract (Sigma) and 0.05% l-cysteine (Sigma) (BHIS) or in MM (38). Strains were grown under anaerobic conditions at 37°C in a Modular Atmosphere Control System 500 (Don Whitney Scientific). All media underwent at least 4 h preequilibration prior to inoculation. Thiamphenicol (Tm) was used at 15 μg/ml to ensure retention of the reporter plasmids. Transformant E. coli strains were routinely grown at 37°C in Luria-Bertani (LB) agar or broth with 25 or 12.5 μg/ml chloramphenicol, respectively.

**TABLE 1:** Strains and plasmids used in this study

Strain or plasmid	Relevant feature(s)	Source or reference
*C. difficile* strains		
630Δ*erm*	Erythromycin sensitive strain of 630	42
630Δ*erm* P*_hpdB_*-SNAP	630Δ*erm* with plasmid P*_hpdB_*-SNAP	This study
630Δ*erm* P*_fdx_*-SNAP	630Δ*erm* with plasmid P*_fdx_*-SNAP	This study
630Δ*erm* P*_hpdB_-gusA*	630Δ*erm* with plasmid P*_hpdB_-gusA*	This study
630Δ*erm* P*_hpdB_-phoZ*	630Δ*erm* with plasmid P*_hpdB_-phoZ*	This study
630Δ*erm* P*_fdx_-phoZ*	630Δ*erm* with plasmid P*_fdx_-phoZ*	This study
CDIP217	630Δ*erm sigL*::*erm*	28
630Δ*erm sigL*::*erm* P*_hpdB_*-SNAP	630Δ*erm sigL*::*erm* with plasmid P*_hpdB_*-SNAP	This study
		
Plasmids		
P*_hpdB_*-SNAP	pMTL84151 plasmid carrying a SNAP-tag under the control of the *hpdBCA* promoter region	26; this study
P*_hpdB_-gusA*	pMTL84151 plasmid carrying *gusA* under the control of the *hpdBCA* promoter	This study
P*_hpdB_-phoZ*	pMTL84151 plasmid carrying *phoZ* under the control of the *hpdBCA* promoter	This study
P*_fdx_*-SNAP	pMTL84153 carrying SNAP-tag under control of the *fdx* promoter	This study
P*_fdx_-phoZ*	pMTL84153 carrying *phoZ* under control of the *fdx* promoter	This study
P*_hpdB_*-SNAP P1mut	pMTL84151 plasmid carrying a SNAP-tag under the control of the *hpdBCA* promoter region with a mutation of the P_1_ site from TAT to TGC	This study
P*_hpdB_*-SNAP P2mut	pMTL84151 plasmid carrying a SNAP-tag under the control of the *hpdBCA* promoter region with a mutation of the P_2_ site from TAT to TGC	This study
P*_hpdB_*-SNAP P1mut P2mut	pMTL84151 plasmid carrying a SNAP-tag under the control of the *hpdBCA* promoter region with mutation in both the P_1_ and P_2_ sites from TAT to TGC	This study
P*_hpdB_-phoZ*ΔIR	630Δ*erm* with plasmid P*_hpdB_-phoZ* with the inverted repeat upstream of *hpdB* removed	This study
P*_hpdB_-phoZ*Δ5′IR	630Δ*erm* with plasmid P*_hpdB_-phoZ* with the 5′ arm of the inverted repeat upstream of hpdB removed	This study
pMC358	Plasmid carrying *phoZ*	15
pRPF185	Plasmid carrying *gusA*	25

### Strain and plasmid construction.

All oligonucleotides used in this study are listed in Table 2. The P*_fdx_*-SNAP plasmid was constructed using a G-block of the SNAP coding sequence (Integrated DNA Technology), amplified by PCR (for the oligonucleotides, see Table 2), to add restriction sites NdeI and SacI and was ligated into the multiple cloning site (MCS) of pMTL84153. To generate the P*_hpdBCA_*-SNAP plasmid, a 402-bp fragment from position −399 to +3 (ATG) of the *hpdB* start codon corresponding to the promoter region of *hpdBCA* (Fig. 1) was amplified from 630Δ*erm* genomic DNA, while the SNAP-tag was amplified from P*_fdx_*-SNAP. These two fragments were joined by SOE-PCR to create an in-frame construct, before cloning into the MCS of pMTL84151 using NdeI and SacI restrictions enzymes and T4 ligase. To construct plasmids carrying P*_hpdB_-phoZ* or P*_hpdB_-gusA*, the SNAP-tag with replaced either *gusA* or *phoZ* amplified from pRPF185 (25) and pMC358 (15), with an inverse PCR to remove the SNAP-tag before the addition of PCR amplified *gusA* or *phoZ* using the NEbuilder HiFi assembly system (NEB). To generate the P*_fdx_-phoZ* construct, the backbone of pMTL84153 was amplified by inverse PCR and the insert, *phoZ*, was amplified from pMC358 and then assembled using an NEbuilder HiFi assembly system (NEB).

**TABLE 2:** Oligonucleotides used in this study

Primer	Sequence (5′–3′)	Purpose
oligoAFP316	TTCGTATGGATCCTCCTTACCCAAGTCCTGGTTTC	Cloning of SNAP-tag in to pMTL84153 to create P*_fdx_*-SNAP; forward primer
oligoAFP325	AGTTCACATATGGATAAAGATTGTGAAATG	Cloning of SNAP-tag in to pMTL84153 to create P*_fdx_*-SNAP; forward primer
hpdB_SNAP_For_P5	GGAAGAAATGGATAAAGATTGTGAAATGAAGAGAAC	Amplification of SNAP-tag; forward primer
SNAP_V_rev_P6	CCCGGGTACCGAGCTCGAATTTACCCAAGTCCTGGTTTC	Amplification of SNAP-tag and for SOE-PCR; reverse primer
hpdB_V_For_P3	CCATATGACCATGATTACGAAGATCTGAATTCGATAGGG	Amplification of *hpdBCA* promoter region and for SOE-PCR; forward primer
hpdB_SNAP_Rev_P4	AATCTTTATCCATTTCTTCCCCTCCTTAATC	Amplification of *hpdBCA* promoter region; reverse primer
*gusA*-F-P	TTACGTCCTGTAGAAACCCC	Amplification of *gusA* coding sequence; forward primer
*gusA*-R-P	TCATTGTTTGCCTCCCTG	Amplification of *gusA* coding sequence; reverse primer
*gusA* vector forward	ATTCGAGCTCGGTACCCG	Inverse PCR of p*hpdBCA*-SNAP for construction of p*hpdBCA-gusA*; forward primer
*gusA* vector reverse	CATTTCTTCCCCTCCTTAATC	Inverse PCR of p*hpdBCA*-SNAP for construction of p*hpdBCA-gusA*; reverse primer
PhoZ hpdB Vec F	AAAAGCAGAAATTCGAGCTCGGTACCCG	Inverse PCR of p*hpdBCA*-SNAP for construction of p*hpdBCA-phoZ*; forward primer
PhoZ hpdB Vec R	ACATTGACGGCATTTCTTCCCCTCCTTAATCTTTC	Inverse PCR of p*hpdBCA*-SNAP for construction of p*hpdBCA-phoZ*; reverse primer
PhoZ F	GGAAGAAATGCCGTCAATGTATGGGTAG	Amplification of *phoZ* coding sequence; forward primer
PhoZ R	GAGCTCGAATTTCTGCTTTTTCTTCATTTTG	Amplification of *phoZ* coding sequence; reverse primer
84153 PhoZ F	AAAAGCAGAAGGATCCTCTAGAGTCGAC	Inverse PCR of pMTL84153 with overhangs for *phoZ;* forward primer
84153 PhoZ R	ACATTGACGGCATATGTAACACACCTCC	Inverse PCR of pMTL84153 with overhangs for *phoZ;* reverse primer
PhoZ 84153 F	GTTACATATGCCGTCAATGTATGGGTAG	Amplification of *phoZ* from pMC358 with overhangs for ligation in to pMTL84153; forward primer
PhoZ 84153 R	TAGAGGATCCTTCTGCTTTTTCTTCATTTTG	Amplification of *phoZ* from pMC358 with overhangs for ligation in to pMTL84153; reverse primer
hpdB P1 SDM F	TTTGCACTAATTATAGAAAGATTAAGGA	For mutation of the P_1_ site on any of the reporter plasmids; forward primer
hpdB P1 SDM R	TAGTCGAAAACTTTTTAAGAATGAAAAA	For mutation of the P_1_ site on any of the reporter plasmids; reverse primer
hpdB P2 SDM F	TTCTGCAGAAAGATTATTTTAAAAAGT	For mutation of the P_2_ site on any of the reporter plasmids; forward primer
hpdB P2 SDM R	TTCTCGAGAAAAAATTAAACTTGAA	For mutation of the P_2_ site on any of the reporter plasmids; reverse primer
hpdB IR Rem F	TTTCATTCTTAAAAAGTTTTATACTAATTATAGAAAG	Removal of entire inverted repeat upstream of *hpdB*; forward primer
hpdB 5 IR Rem F	TAATATACCCTTTTTTTTCATTCTTAAAAAG	Removal of 5′ arm of inverted repeat upstream of *hpdB*; forward primer
hpdB IR Rem R	hpdB IR Rem R	Removal of 5′ arm or entire inverted repeat upstream of *hpdB*; reverse primer

Transformation of chemically competent (NEB5α; NEB) or electrocompetent (Top10; Thermo Fisher) cells was performed according to the manufacturer’s instructions. Reporter constructs were checked by sequencing, and the corresponding plasmids were transferred by electroporation into E. coli CA434 cells used for conjugation into C. difficile (39). Transconjugants were selected on BHIS with C. difficile supplement (CC; Oxoid) and Tm (15 μg/ml) (CCTm) plates. Colonies were restreaked once more on BHIS CCTm plates to ensure plasmid transfer and stored at −80°C for future use. Plasmid maps were drawn with Snapgene software.

### Reporter comparison.

The strains containing different fusions were grown overnight in BHIS with thiamphenicol (15 μg/ml). A 2.5-ml portion of the overnight sample was added to 7.5 ml of MM (38) containing 100 mM glucose and grown for 3 h. After 3 h of growth, the cultures were pelleted and washed using 5 ml of fresh MM before repelleting. Pellets were resuspended in 1 ml of MM, and 100 μl of this suspension was added to 10 ml of each of the following test conditions: MM only, MM plus 2 mg/ml *p*-HPA, and MM plus 0.4 mg/ml tyrosine. Strains were grown in biological triplicates for 4 h before processing required for each of the reporters as described below:

### SNAP-tag reporter.

Next, 1 ml of culture was used for the OD_590_ reading to assess growth. A 5-ml portion was removed for testing the SNAP-tag, the SNAP-tag substrate TMR-Star (NEB) was added at 0.2 nM to 5-ml portions of cultures, and the mixture was incubated in the dark for 30 min at 37°C. These samples were pelleted and washed with 5 ml of phosphate-buffered saline (PBS). After resuspension of the pellet in 600 μl of PBS, these samples were transferred to a Lysis Matrix B tube (MP Biomedicals) and lysed for 40 s at 6.0 m/s twice using a FastPrep-24 Classic instrument (MP Biomedicals). After centrifugation, the supernatant was used to quantify SNAP-tag production via SDS-PAGE (10% Bis-Tris protein gel). Gels were imaged using Typhoon Trio Variable Mode Imager System (GE Healthcare), and fluorescence was detected at 580 nm. Analysis was carried out using ImageQuant TL image analysis software. Expression was detected by gating bands corresponding to the SNAP-tag and a control that was subtracted by measuring pixels from an equal size gate directly above that of the SNAP-tag band. Background controls were calculated based on the background pixel volume/OD_590_; the average was taken for all samples per experiment to give an average background fluorescence that was considered to be the limit of detection (LOD). SNAP-tag production was quantified according to the following formula: product intensity = pixel volume/OD_590_. These experiments were undertaken with at least triplicate biological replicates. The expression from the SNAP-tag fusion with the upstream region of *hpdBCA* containing the wild-type promoter region or deletion of putative promoters, P_1_ and/or P_2_, was carried out as above; however, all strains were grown in MM in the presence of 2 mg/ml *p*-HPA.

### Scaled-up SNAP-tag.

The mutated P_1_ promoter was tested further with initial growth and 3-h growth steps in BHIS and MM as described above; however, 100 μl was inoculated in to 45 ml of MM, without glucose, in each of the test conditions: MM only, MM plus 2 mg/ml *p*-HPA, and MM plus 0.4 mg/ml tyrosine. After 24 h of growth, 1 ml was removed for OD_590_ determination to assess growth, and the remaining culture was pelleted and resuspended in 4 ml of fresh MM. Prepared triplicate biological samples underwent SNAP-tag assay testing as described above. To investigate a putative role of SigL (σ^54^) in *hpdBCA* expression, the SNAP-tag reporter fusion was transferred into the 630Δ*erm sigL*::*erm* strain (28). Due to the growth limitations of strain 630Δ*erm sigL*::*erm* strain in minimal medium, BHIS supplemented with 100 mM glucose (BHISG) was chosen for all analyses undertaken. For these experiments, initial overnight cultures were carried out in 10 ml of BHISG before 100 μl of the overnight culture was inoculated into 45 ml of fresh BHISG and grown for 24 h before pelleting and resuspension in 4 ml of BHISG. Prepared biological triplicate samples underwent SNAP-tag assay testing as described above.

### *gusA* reporter.

Triplicate biological samples were prepared as described above. After 4 h of growth in MM, 1 ml of the culture was removed to detect the OD_590_, and 1.5-ml samples were pelleted and frozen for later testing as described previously by Mordaka and Heap (31). The OD_590_ after 4 h was used to normalize for growth so that growth was taken in to account in the same way as the other reporters. Testing was carried out by resuspension of the pellets in 0.8 ml of buffer Z (60 mM sodium phosphate dibasic heptahydrate, 10 mM KCl, 40 mM sodium phosphate monobasic, 1 mM magnesium sulfate heptahydrate; the pH was adjusted to 7.0, and 50 mM 2-mercaptoethanol was added freshly). Then, 600 μl was transferred to a fresh microcentrifuge tube, and 6 μl of toluene was added, followed by vortexing for 1 min and incubation on ice for 10 min. Samples were heated at 37°C for 30 min with the microcentrifuge caps open. After heating, 120 μl of 6 mM *p*-nitro phenyl-β-d-glucuronide in buffer Z was added to start the assay reaction, followed by incubation for 30 min at 37°C, before the addition of 1 M sodium carbonate to stop the reaction. Samples were centrifuged for 10 min at 10,000 rpm, and supernatants were transferred to a 24-well plate. Absorbance was measured at 405 nm using a Spectramax M3 Multi-Mode plate reader (Molecular Devices) with *gusA* activity calculated using the following formula: final OD_405_/culture OD_590_.

### *phoZ* reporter.

Triplicate biological samples were prepared as described above with sample testing carried out according to the method of Edwards et al. (15). After 4 h of growth in MM, 1 ml of the culture was taken for OD_590_ measurement, and 2-ml samples were pelleted and frozen for assays. The OD_590_ after 4 h was used to ensure growth was taken into account identically to the other reporters. Testing was carried out by resuspension of the pellets in 500 μl of cold wash buffer (10 mM Tris-HCl [pH 8], 10 mM magnesium sulfate) prior to pelleting and resuspension in 1 ml of assay buffer (1 M Tris-HCl [pH 8], 0.1 mM zinc chloride). Then, 500 μl of the cell suspension was added to 300 μl of fresh assay buffer, 50 μl of 0.1% SDS, and 50 μl of chloroform. Samples were vortexed for 1 min, incubated in a water bath at 37°C for 5 min, and then chilled on ice for 5 min. Samples were then preheated to 37°C, and 100 μl of 0.4% *p*-nitrophenyl phosphate in 1 M Tris-HCl (pH 8) was added. The samples were incubated at 37°C until a yellow color developed, at which point 100 μl of stop solution (1 M potassium phosphate monobasic) was added and the time taken for the color to develop was recorded. Samples underwent pelleting by centrifugation at maximum speed for 5 min, and the supernatants were transferred to a spectrophotometer cuvette. The optical densities were measured at 420 and 550 nm using a Spectramax M3 Multi-Mode plate reader with activity quantified as follows: [OD_420_ – (OD_550_ × 1.75)] × 1,000/*t* (min) × OD_590_ × cell volume (ml).

### HPLC.

Supernatants were obtained from C. difficile cultures grown for 10 h in MM and in MM supplemented with either 2 mg/ml *p*-HPA or 0.4 mg/ml tyrosine. The MM was altered from that used above to contain defined amino acids as described by Karasawa et al. (40), with the exception of glycine being increased to 0.4 mg/ml, and tyrosine was only included in samples as indicated. In addition, glucose was not included to ensure maximum usage of amino acids such as tyrosine. Supernatants were filter sterilized using 0.2-μm filters and stored at −80°C. Defrosted culture supernatants were mixed in a 1:1 ratio with methanol-water, transferred to HPLC tubes, and processed immediately by HPLC. Separations were performed utilizing an Acclaim 120 (Thermo Fisher) C_18_ 5-μm analytical (4.6 by 150 mm) column and a mobile phase consisting of ammonium formate (10 mM [pH 2.7]) and menthol (vol/vol; 40:60) at a flow rate of 2 ml/min. Tyrosine, *p*-HPA, and *p*-cresol were detected using a photodiode array detector (UV-PDA; DAD 3000) set at 280 nm. The peak identity was confirmed by measuring the retention times of commercially available tyrosine, *p*-HPA, and *p*-cresol and determination of the absorbance spectra using the UV-PDA detector. A calibration curve of each compound was generated by Chromeleon (Dionex Software) with known amounts of the reference standards (0 to 100 mg/ml) in methanol-water (1:1 [vol/vol]) injected onto the column, from which the concentrations in the samples were determined. Samples from three independent biological replicates were analyzed compared to medium controls and standard curves. The data were analyzed in GraphPad Prism7, and statistical analysis was performed in Stata16 using linear regression analysis *P* < 0.05 was considered statistically significant.

### RNA extractions and qRT-PCR.

Total RNA was isolated from C. difficile 630Δ*erm* and *sigL* mutant strains grown in BHISG medium to an OD_590_ of 1.2 ± 0.2. RNAprotect (Qiagen) was added to exponential or early-stationary-phase cultures, cells were pelleted at 4°C, and pellets were stored at −80°C. After defrosting on ice, the pellets were processed using the RNAPro extraction kit (MP Biomedicals). Samples were DNase treated twice with 10 U of Turbo DNase (Invitrogen) and 40 U of RNase inhibitor (NEB). Samples were purified by using a Qiagen RNase kit. Samples were checked for DNA contamination by PCR. RNA integrity was determined by using an Agilent RNA Nano Bioanalyser Chip (Agilent), followed by cDNA synthesis via reverse transcription and quantitative real-time PCR analysis. cDNA was synthesized from 200 ng of total RNA. Random primers were annealed to the RNA by heating at 80°C for 5 min, followed by cooling slowly to room temperature. A 10 mM concentration of each deoxynucleoside triphosphate and 200 U of reverse transcriptase III in 1× RT buffer (Thermo Fisher) were added in reaction mixtures to a final volume of 25 μl. Reverse transcriptase-negative samples were included as controls (RT–). Real-time quantitative PCR was performed in a 20-μl reaction volume containing 10 ng (for *hpdC*) or 200 pg (for 16S rRNA) of cDNAs, along with 12.75 μl of the SYBR PCR master mix (Applied Biosystems), and 10 μM concentrations of each gene-specific primer. Amplification and detection were performed by using an ABI 7500 thermocycler. In each sample, the quantity of cDNA of a gene was determined by subtracting the RT– and then normalized to the quantity of cDNA of the 16S rRNA gene. The relative change in gene expression was recorded as the ratio of normalized target concentrations (determined using the threshold cycle [ΔΔ*C_T_*] method) as previously described (27). The relative change in gene expression was recorded as the ratio of normalized target concentrations (ΔΔ*C_T_*), expressed as 2^–ΔΔ^*^CT^* (41) for triplicate technical and duplicate biological replicates. The data were analyzed in GraphPad Prism7, and statistical analysis was performed in Stata16 using linear regression analysis (*P* < 0.05 was considered statistically significant).

### Statistical analysis.

The expression data from the transcriptional reporter fusions were analyzed for significant differences in expression under different conditions. The data were transformed using log_10_ to approximate a normal distribution, and then linear regression analysis was used to determine significant differences (i) between growth conditions, including the addition of tyrosine and *p*-HPA, compared to the minimal medium control; (ii) between fold changes in expression between reporters and in comparison to qRT-PCR in the presence of *p*-HPA; (iii) between the wild-type and mutated −10 promoter regions; (iv) between the wild-type and the *sigL* mutant; and (v) in the fold change (2^–ΔΔ^*^CT^*) between the control media and the addition of *p*-HPA, as well as between the wild-type and the *sigL* mutant strain. HPLC analysis was analyzed by linear regression to determine whether there were any differences in the concentration of tyrosine, *p*-HPA, and *p*-cresol detected in the cultures, and the correlation between the *phoZ* activity and *p*-HPA concentration was analyzed using linear regression. Statistical differences are indicated in the figures. The coefficient of variance (COV) indicates whether the difference between the test samples is higher (positive number) or lower (negative number) than the reference. Analysis was performed using Stata15 (StataCorp); statistical test summaries are available in File S1 in the supplemental material.

## Supplementary Material

Supplemental file 1

Supplemental file 2
