# Supplementary material for: Clostridioides difficile para-Cresol Production Is Induced by the Precursor para-Hydroxyphenylacetate
Source: J Bacteriol. 2020 Aug 25;202(18):e00282-20. doi: 10.1128/JB.00282-20 (PMC7925072; doi:10.1128/JB.00282-20)
Supplement: Supplemental file 2 [file JB.00282-20-s0002.pdf]

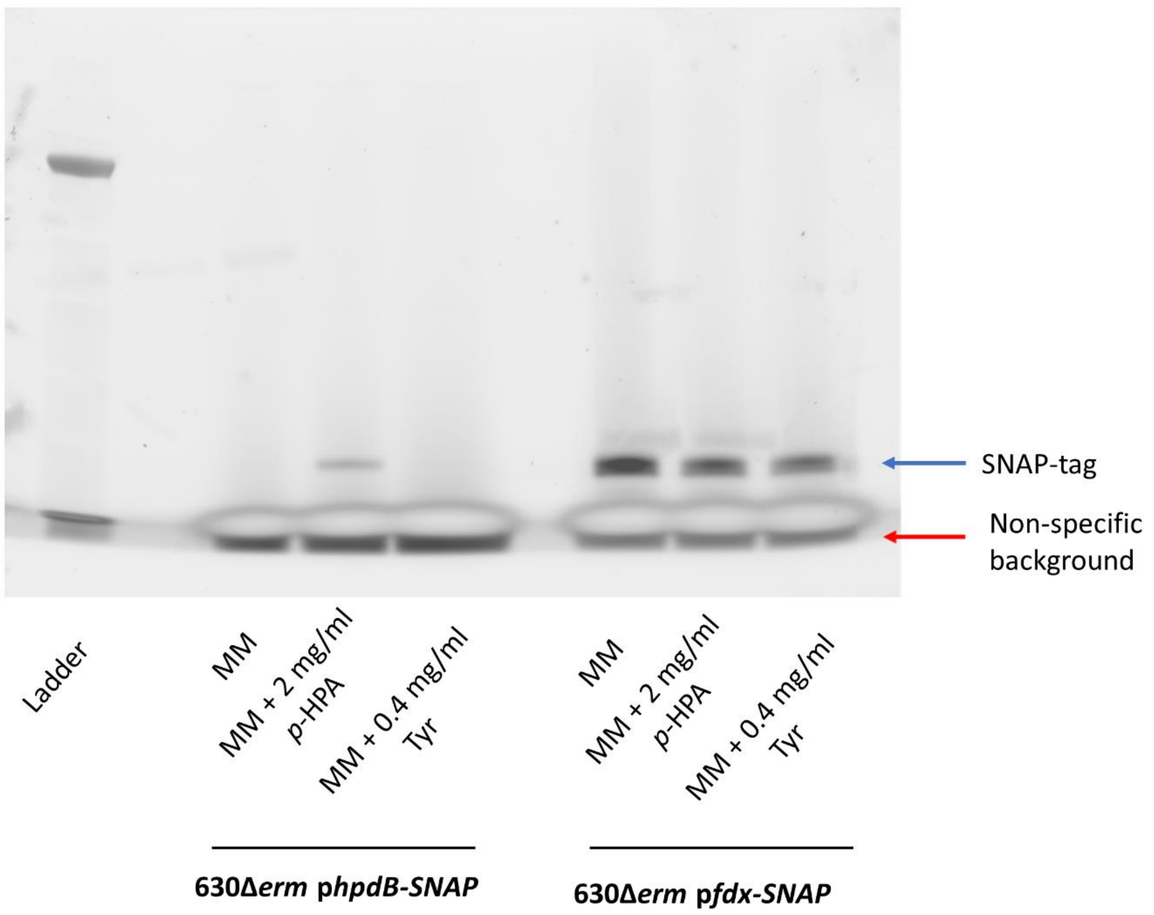

Figure S1. Uncropped SNAP-tag gel. Uncropped SNAP-tag gel. Strains 630Δerm P_{hpdB}-SNAP and 630Δerm P_{fdx}-SNAP were grown for four hours in MM alone, or MM supplmeted with 2 mg/ml *p*-HPA or 0.4 mg/ml Tyrosine. Samples were processed with the fluorescent substrate TMR-Star prior to being run on an SDS-PAGE gel and imaged using a Typhoon Trio Variable Mode Imager System (GE Healthcare) with fluorescence detected at 580 nm. Background fluorescence is labelled with a red arrow. The blue arrow indicates the SNAP tag signal.
